# The Effect of COVID-19 on Lung Cancer Screening

**DOI:** 10.7759/cureus.68986

**Published:** 2024-09-09

**Authors:** Nidhi S Thiruppathi, Nicole C Humphries, Samantha Johnson, Laura Tunison, Andrew Pippas

**Affiliations:** 1 Research, Mercer University School of Medicine, Columbus, USA; 2 Medical School, Mercer University School of Medicine, Columbus, USA; 3 Hematology and Oncology, Piedmont Healthcare, Columbus, USA

**Keywords:** cancer screening, coronavirus, covid-19, lcs, ldct, low-dose computed tomography, lung cancer, nosocomial infection, pandemic, preventative medicine

## Abstract

Background

During the COVID-19 pandemic, many hospitals suspended non-essential medical procedures to reduce transmission and prioritize personal protective equipment (PPE) for COVID-19 patients. Hospitals that continued these procedures faced uncertainty about patient attendance. Multiple factors could explain a decline in patient attendance during the pandemic, including patients' reluctance to risk COVID-19 exposure in the hospital or their own illness requiring self-isolation. This study aimed to compare attendance rates of lung cancer screenings (LCS) before and during the pandemic. Unlike previous studies conducted on this research topic, the current study documents that the John B. Amos Cancer Center continued LCS throughout the pandemic. The alternative hypothesis was that there would be a decrease in the percentage of LCS performed during the pandemic period due to fear of nosocomial transmission.

Materials and methods

Data for 2,582 scheduled LCS were retrospectively analyzed on Microsoft Excel 2022 (Microsoft Corporation, Redmond, Washington) from 2018 to 2021. For analysis purposes, 2018 and 2019 were considered pre-COVID years, while 2020 and 2021 were considered COVID years. The average percentage attended was calculated for each year and the standard deviation of that year's percentage. The percentage of patients seen each month was averaged during pre-COVID and COVID years. The p-value was calculated by comparing the average attendance percentage for each month in the pre-COVID and COVID years. A p-value <0.05 was considered significant.

Results

From 2018 to 2021, over 300 more people were scheduled during the COVID years. Although the percentage seen remained consistent throughout the years, there was an increase in both patients scheduled and seen. The results revealed an insignificant difference in LCS attendance between pre-COVID and COVID years, confirming the importance of their continuation.

Conclusion

The alternative hypothesis was rejected due to no significant difference in attendance percentage between the pre-COVID and COVID years. Further direction of this study may include monitoring the trend of LCS attendance during post-pandemic years as the transmission rates continue to change.

## Introduction

Cancer mortality rates in the United States have declined in the last 30 years by over 30%. Even with this substantial decrease, cancer has remained the second leading cause of mortality in the country, competing with heart disease [1]. Lung cancer, specifically, is responsible for approximately 20% of cancer deaths worldwide. The National Cancer Institute's Surveillance, Epidemiology, and End Results reports 229,000 new cases of lung cancer in 2020 alone [2]. However, if caught early, lung cancer can be managed and potentially cured.

The most effective modality of lung cancer screening (LCS) is low-dose computed tomography (LDCT) [3]. This mechanism can help diagnose lung cancer before severe symptoms present. Symptoms of lung cancer include persistent coughing, chest pain, dyspnea, wheezing, hemoptysis, fatigue, and unintended weight loss. Patients may even present with infectious symptoms, including recurrent episodes of pneumonia and mediastinal lymphadenopathy [4]. Criteria are available to guide suitable populations before symptoms occur. The US Preventive Services Task Force published criteria stating annual LCS is recommended for adults 55 to 74 years old who have a 30-pack-year smoking history and are currently smoking or quit within the last 15 years [5]. The National Lung Screening Trial established these guidelines and stated that the occurrence of the screenings reduced lung cancer mortality by 20% [6].

The COVID-19 pandemic has impacted every medical institution worldwide. This global outbreak has taken the lives of over one million Americans. The virus is transmitted by respiratory droplets and airborne particles, causing fear of nosocomial contamination during the virus's onset in March 2020. As a result, many hospitals in the United States decided to temporarily halt elective procedures in hopes of reducing COVID-19 transmission while also allocating protective personal equipment (PPE) to physicians treating patients infected with the virus [7]. The question of what is considered an "elective" procedure quickly arose within the medical community.

During a White House Task Force press briefing in March 2020, the Centers for Medicare & Medicaid Services announced that elective procedures would be delayed during the pandemic. This was to limit exposure to the virus and allocate protective equipment, beds, and ventilators to patients infected by the COVID-19 virus. Tiers of which procedures were considered elective were published; Tier One consisted of low-acuity services, and Tier Three consisted of high-acuity treatment that was to be continued [8]. LCS were labeled a Tier One service. Delaying elective procedures remained ultimately under the discretion of hospitals and clinics, despite the federal recommendation.

This study investigated whether the percentage of LCS attendance altered during the pandemic. The study population included patients at the John B. Amos Cancer Center in Columbus, Georgia. This organization chose to continue LCS during the COVID-19 pandemic. It was hypothesized that the percentage of LCS performed during the pandemic period would decrease due to fear of nosocomial transmission.

## Materials and methods

The nurse navigator for LCS gathered the data at the John B. Amos Cancer Center in Columbus, Georgia. Data for 2,582 scheduled LCS were retrospectively analyzed on Microsoft Excel 2022 (Microsoft Corporation, Redmond, Washington) from 2018 to 2021. The goal was to interpret if the percentage of patients attending their LCS was affected by COVID-19. A two-sample t-test was used to analyze if a significant difference was present between LDCT attendance rates for pre-COVID and COVID years. ANOVA (analysis of variance) was used to analyze monthly data from 2018 to 2021 to see if there are significant differences in monthly attendance rates. For analysis purposes, 2018 and 2019 were considered pre-COVID years, while 2020 and 2021 were considered COVID years. The data included the patient's date of birth, returning/new patient status, smoking status, smoking cessation enrollment, date of service, screening findings, incidental findings, and physician referral status. The screening findings also indicated if a patient missed their appointment by stating if the patient was a no-show or cancellation. The average age, number of smokers, number of no-shows, and percentage of patients who attended their screening were calculated from these data by year.

The average percentage attended was also calculated for each year, as well as the standard deviation of that year's percentage. The percentage of patients seen each month was averaged during pre-COVID and COVID years. The total number of no-shows was shown on a line graph comparing each month of each year. The total percentage of LCS attendance was displayed on a line graph comparing each month consecutively from 2018 to 2021. The p-value was calculated by comparing the average attendance percentage for each month in the pre-COVID and COVID years. A p-value <0.05 was considered significant.

## Results

While analyzing the total number of patients scheduled from 2018 to 2021, it was noted that over 300 more people were scheduled during the COVID years (Table 1). Although the percentage seen remained consistent throughout the years, there was an increase in both patients scheduled and seen.

**Table 1:** Analysis of patients scheduled for lung cancer screening at the John B. Amos Cancer Center between 2018 and 2021

Year	2018	2019	2020	2021	Total
Total scheduled	533	582	701	766	2582
No-show	97	134	152	139	522
Percent seen	81.6	78.8	77.4	81.4	79.8
Average age (years)	69	69	68	66	68
Current smokers	319	357	427	479	1582

**Figure 1:**
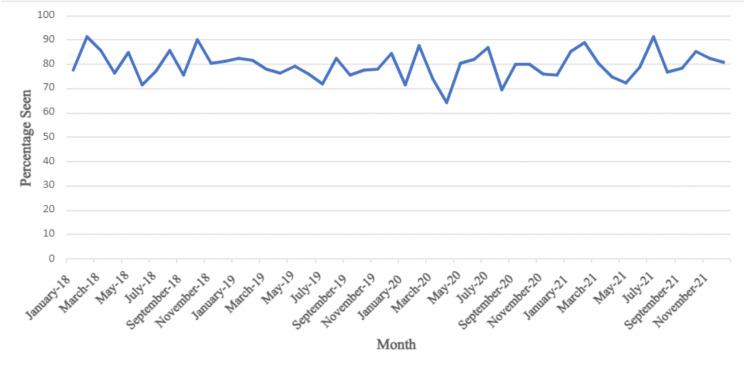
Total percentage of lung cancer screening attendance at the John B. Amos Cancer Center between 2018 and 2021

The results indicated that the lowest LCS attendance recorded from 2018 to 2021 was in April 2020, with an attendance rate of 64.3%, compared to the average LCS attendance rate of 79.8%. However, the overall average of LCS attendance during the COVID years (2020-2021) was calculated at 79.4%. This was compared to the overall average of LCS attendance during the pre-COVID years (2018-2019), which was calculated at 80.1% (Figure 1). The p-value was 0.92

**Figure 2:**
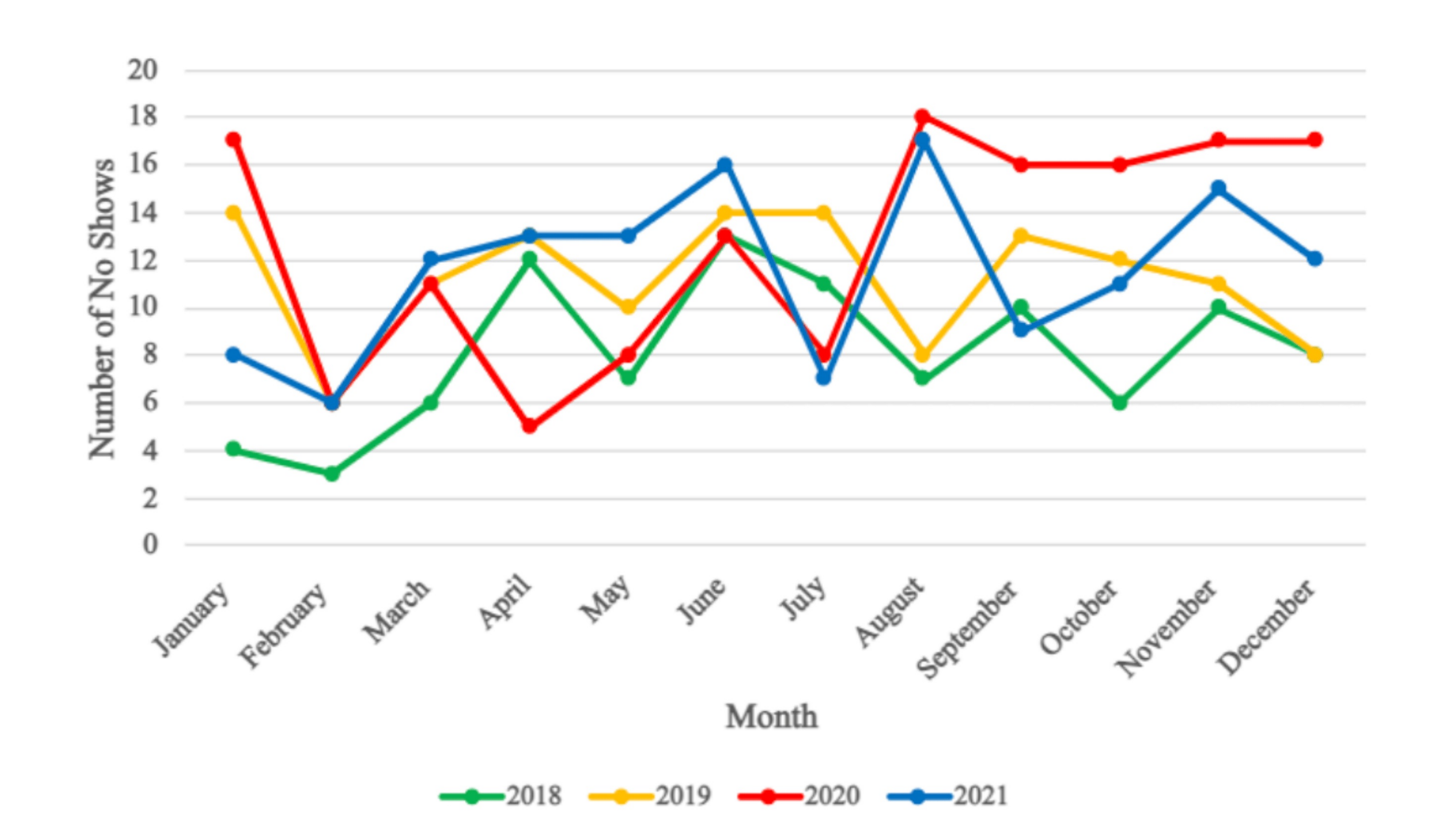
Total number of patients who did not attend their scheduled lung cancer screening, comparing 2018, 2019, 2020, and 2021

Regarding no-shows, the pre-COVID years had a total of 97 and 134 no-shows, respectively. However, in 2020, the number of no-shows increased to 152 (Figure 2). Compared to the pre-COVID years, the number of no-shows remained slightly elevated in 2021, at 139. As for the individual months, August 2020 had the most no-shows recorded, with 18 patients who did not show up for the LCS.

## Discussion

Lung cancer staging is dependent on the TNM staging system, with T referring to tumor size, N referring to lymph node involvement, and M referring to the presence of metastatic disease [4]. Regarding prognosis, survival is largely determined by the stage of disease and treatment modality. If the disease is still localized within the lungs, the five-year survival rate is 56%. This is in stark contrast to metastatic lung tumors, with a five-year survival rate of 5%. Additionally, only approximately 10% of people are diagnosed with lung cancer at an early stage, highlighting the role of prompt screening [9].

The decrease in attendance rates in April 2020 correlates with the first full month of the pandemic shutdown. As the Guidelines from the Centers for Medicare & Medicaid Services were submitted in mid-March, it is suspected that the organization may have even cancelled these appointments in an attempt to make an informed decision on how they would like to proceed with LDCT, deciding whether cancelling or continuing was more beneficial for their population. Along with an institution's decision to halt screening, other factors influenced screening attendance rates. Patients had to decide whether the risk of nosocomial COVID-19 infection outweighed the perceived benefits of LCS. A decrease in attendance during this time would have supported this analysis. Gibney et al. presented arguments supporting both the continuation and discontinuation of LCS during the pandemic. These authors took a stronger stance supporting continued screenings, reaffirming that nosocomial transmission is rare when proper precautions and PPE are utilized. Their research also found that many of the lung screening resources did not subtract from the resources used to treat patients who had COVID-19 [7]. LDCT is a quick procedure that involves minimal human interaction. With both the patient and the staff members wearing masks, little PPE is withdrawn from the front lines while they are battling the coronavirus. These studies are also typically performed in a separate facility, avoiding interactions with hospitalized patients with COVID-19.

The results from the current research study showed that the percentage of LCS remained consistent from 2018 to 2021, with an increase in LCS scheduled in 2020 and 2021. With a p-value of 0.92, there was no significant difference in the percentage of patients seen before and during the COVID-19 pandemic, accepting the null hypothesis. We suspect that the increase in LDCT attendance was secondary to the increased awareness of one's health. This virus has the most detrimental effects on immunocompromised individuals or individuals with respiratory compromise. We speculate that people wanted to understand their own health status, as there was already so much uncertainty surrounding the virus's transmission and prognosis. This could help them to better understand their health category regarding precautions.

The continuance of LCS attendance forces hospitals to consider the future consequences of halting such procedures during future widespread outbreaks. Considerations would depend on the type of virus, its contagion level, and the transmission rate. Those risk factors must be weighed against the benefit that the procedures, such as screenings, would have on the patient's mortality.

## Conclusions

The alternative hypothesis was rejected since there was no significant difference between the percentage of attendance between the pre-COVID and COVID years. Further directions can include analysis of 2022 data to see if the percentage of LCS attendance at the John B. Amos Cancer Center remained unchanged, thus aligning with the trends found in this research study.
